# Circulating perilipin 2 levels are associated with fat mass, inflammatory and metabolic markers and are higher in women than men

**DOI:** 10.18632/aging.202840

**Published:** 2021-03-17

**Authors:** Maria Conte, Aurelia Santoro, Salvatore Collura, Morena Martucci, Giuseppe Battista, Alberto Bazzocchi, Cristina Morsiani, Federica Sevini, Miriam Capri, Daniela Monti, Claudio Franceschi, Stefano Salvioli

**Affiliations:** 1 Department of Experimental, Diagnostic and Specialty Medicine (DIMES), University of Bologna, Bologna, Italy; 2 Interdepartmental Center “Alma Mater Research Institute on Global Challenges and Climate Change (Alma Climate)”, University of Bologna, Bologna, Italy; 3 Diagnostic and Interventional Radiology, IRCCS Istituto Ortopedico Rizzoli, Bologna, Italy; 4 Department of Experimental and Clinical Biomedical Sciences “Mario Serio”, University of Florence, Florence, Italy; 5 Laboratory of Systems Medicine of Healthy Aging and Department of Applied Mathematics, Lobachevsky University, Nizhny Novgorod, Russia

**Keywords:** circulating perilipin 2, body composition, fat mass, leptin

## Abstract

Perilipin 2 (PLIN2) is a protein involved in lipid storage and metabolism in non-adipose tissues. Detectable levels of circulating PLIN2 (cPLIN2) have been reported to be associated with some types of cancer, but no systematic analysis of age-related modifications in cPLIN2 levels has ever been performed. We measured serum cPLIN2 in a group of old people including centenarians in comparison with young subjects and tested possible correlations with parameters of body composition, fat and glucose metabolism, and inflammation. We found that: i. levels of cPLIN2 do not change with age, but women have higher levels of cPLIN2 with respect to men; ii. cPLIN2 levels strongly correlate to BMI, as well as fat and lean mass; iii. cPLIN2 levels strongly correlate with the proinflammatory adipokine leptin. Due to the adipogenic activity of leptin, it is hypothesized that cPLIN2 is affected and possibly regulated by this pleiotropic adipokine. Moreover, these results suggest that cPLIN2 (possibly together with leptin) could be assumed as a proxy for body adiposity.

## INTRODUCTION

Perilipin 2 (PLIN2), also known as adipose differentiation-related protein (ADRP) or adipophilin, is a member of the PAT family, an evolutionary conserved protein family, involved in several intracellular processes, such as lipid metabolism and transport, as well as lipid droplet (LD) formation and stability [1, 2]. PLIN2 is always strictly located at the surface of LDs and, for this reason, it is considered a marker for LDs accumulation [2]. PLIN2 was identified for the first time in pre-adipocytes during their differentiation into adipocytes only as mRNA expression, however, the expression of PLIN2 as protein in pre- and mature adipocytes has never been observed [1, 3, 4]. PLIN2 is expressed in several non-adipose tissues, including skeletal muscle, liver, pancreas and mammary gland, where it plays a role in regulating lipid storage and accumulation [1].

In the last decades, PLIN2 received considerable attention, as it is essential for the normal physiology of the cell. Several *in vitro* and *in vivo* studies, performed in different types of cells, mice and humans, demonstrated that the differential expression of PLIN2 is associated with alterations of intracellular lipid metabolism, deregulation of fatty acid uptake and LD formation, leading to several metabolic disorders and age-related diseases [1]. In previous studies, we observed that high levels of PLIN2 in both humans and mice are associated with skeletal muscle atrophy and sarcopenia [3, 5, 6]. In agreement with these results, it has been demonstrated that in mouse high levels of PLIN2 are associated with several metabolic diseases, such as obesity, diabetes, fatty liver diseases, atherosclerosis and cardiovascular diseases, while the inhibition of PLIN2 prevents or alleviates these pathologies [1, 7–10]. Other *in vitro* studies have demonstrated that increased levels of PLIN2 are present in different types of tumor, suggesting that PLIN2 may play an important role in tumorigenesis [11–14]. Interestingly, it has been reported that PLIN2 is present not only as intracellular protein, but also at circulating level in body fluids, such as urine and plasma. In particular, elevated circulating PLIN2 (cPLIN2) levels were found in plasma or urine of patients with some types of cancer (kidney, colorectal) with respect to healthy subjects [15, 16].

To date, little is known about the modifications of cPLIN2 levels with age and its possible association with health parameters and body composition. This study aimed to analyze the levels of cPLIN2 in two cohorts of subjects, the first composed of subjects of different age (cohort 1), including centenarians, the latter composed of subjects of similar age but with different amount of total fat mass (cohort 2).

## RESULTS

### cPLIN2 levels are higher in old women and positively associated with metabolic parameters and inflammatory markers

In order to study possible age-related modifications of cPLIN2 levels, we first analyzed a group of 189 subjects (Cohort 1). In particular, we compared a group of 35 young subjects (age range 24-39) with 154 old subjects, including centenarians, divided into four groups: 57 OLD1 subjects (age range 60-69); 33 OLD2 subjects (age range 70-79); 26 OLD3 subjects (age range 85-99); 38 centenarians (age range 100-107). The results of cPLIN2, as well as of anthropometric and biochemical parameters are reported in Table 1. cPLIN2 levels are significantly higher in women than men, and this difference becomes more dramatic in the group of old subjects (OLD1 and OLD2), however nonagenarians (OLD3) and centenarians show no difference.

**Table 1:** Anthropometric and biochemical characteristics of Cohort 1.

**Characteristics (mean ± SE)**	**YOUNG (N° 35, age range 24-39)**			**OLD1 (N° 57, age range 60-69)**			**OLD2 (N° 33, age range 70-79)**			**OLD3 (N° 26, age range 85-99)**			**CENTENARIANS (N° 38, age range 100-107)**		
	**18 F**	**17 M**	**p value**	**37 F**	**20 M**	**p value**	**16 F**	**17 M**	**p value**	**13 F**	**13 M**	**p value**	**21 F**	**17 M**	**p values**
cPlin2 (ng/ml)	35.25 ± 4.38	22.63 ± 4.37	0.038	50.46 ± 2.76	30.63 ± 3.43	0.000	67.22 ± 5.39	28.34 ± 4.60	0.000	35.55 ± 7.55	30.89 ± 7.80	n.s.	30.98 ± 5.77	32.07 ± 4.97	n.s.
BMI	21.63 ± 0.49	26.13 ± 1.06	0.001	29.56 ± 0.75	31.72 ± 1.06	n.s.	30.11 ± 1.39	28.66 ± 0.89	n.s.	25.51 ± 0.86	28.31 ± 1.54	n.s.	24.18 ± 0.96	24.47 ± 0.70	n.s.
Waist circumference (cm)	70.79 ± 0.87	85.13 ± 3.60	0.000	95.99 ± 1.78	107.0 ± 2.68	0.001	95.83 ± 3.66	99.62 ± 2.23	n.s.	---	---	n.s.	79.42 ± 2.32	92.40 ± 3.04	0.001
Hip circumference (cm)	98.36 ± 1.28	98.81 ± 2.40	n.s.	107.77 ± 1.70	109.75 ± 2.20	n.s.	110.97 ± 2.85	104.97 ± 2.23	n.s.	---	---	n.s.	93.70 ± 2.24	98.20 ± 2.53	n.s.
Handgrip (kg)	30.18 ± 1.02	47.50 ± 2.5	0.000	23.0 ± 1.03	42.33 ± 2.6	0.024	22.25 ± 1.82	30.33 ± 1.71	0.006	20.17 ± 1.85	17.88 ± 2.32	n.s.	12.15 ± 1.24	18.44 ± 1.19	0.002
Glycaemia (mg/dl)	82.73 1.77	85.83 ± 1.16	n.s.	131.68 ± 6.24	144.15 ± 13.17	n.s.	112.06 ± 9.86	119.06 ± 9.44	n.s.	80.92 ± 4.50	90.38 ± 8.52	n.s.	86.05 ± 2.55	86.41 ± 3.89	n.s.
Total cholesterol (mg/dl)	185.80 ± 7.42	194.0 ± 12.83	n.s.	205.46 ± 5.92	201.10 ± 8.08	n.s.	208.13 ± 7.61	202.29 ± 10.72	n.s.	192.85 ± 9.19	179.92 ± 11.08	n.s.	201.29 ± 11.05	181.59 ± 9.97	n.s.
HDL (mg/dl)	63.0 ± 7.42	55.33 ± 5.70	n.s.	53.70 ± 2.54	48.85 ± 2.28	n.s.	61.69 ± 5.16	50.82 ± 4.50	n.s.	66.62 ± 3.12	56.92 ± 5.55	n.s.	51.29 ± 3.08	47.29 ± 3.28	n.s.
LDL (mg/dl)	111.33 ± 5.21	124.83 ± 9.71	n.s.	126.16 ± 5.4	113.11 ± 5.82	n.s.	113.52 ± 14.12	110.61 ± 12.10	n.s.	104.78 ± 7.91	101.92 ± 9.18	n.s.	120.95 ± 9.28	106.67 ± 11.90	n.s.
Triglycerides (mg/dl)	57.87 ± 4.23	69.83 ± 12.52	n.s.	131.22 ± 19.51	136.9 ± 23.58	n.s.	130.81 ± 9.47	159.47 ± 29.78	n.s.	107.23 ± 10.32	104.38 ± 9.51	n.s.	116.95 ± 6.75	98.94 ± 8.71	0.048
Average number of diseases	0			1.46			1.57			0.84			0.60		
Current diseases				angina pectoris (1), diabetes (36), hepatitis (1), hypercholesterolemia (13), hypertension (32)			angina pectoris (1), diabetes (13), heart failure (1), hepatitis (1), hypercholesterolemia (11), hypertension (24), obliterative arteriopathy (1)			diabetes (2), heart failure (3), hypercholesterolemia (3), hypertension (12), Irregular heart rhythm (2)			Angina pectoris (3), diabetes (3), heart failure (9), hypercholesterolemia (3), hypertension (5)		

Legend: n.s. = not significant.

Since BMI strongly correlates with cPLIN2, in both women (Spearman correlation parameters: ρ=0.572, p<0.001) and men (Spearman correlation parameters: ρ=0.712, p<0.001), further analyses were conducted considering BMI as a covariate. After adjusting for BMI, the difference in cPLIN2 levels by sex was confirmed, in particular women belonging to young, OLD1 and OLD2 groups showed significantly (p<0.001) higher levels of cPLIN2 with respect to men of the same age groups.

cPLIN2 levels appear to change with age, in particular they seem to be higher in women belonging to OLD1 and OLD2 groups with respect to young, nonagenarian, or centenarian subjects (Table 1), however, after adjusting for BMI, the difference between age groups disappeared. An apparent difference was noticed between young and OLD2 males, despite the similarity in BMI values. However, this difference resulted not statistically significant. A partial correlation of cPLIN2 with anthropometric, metabolic and inflammatory parameters after adjusting for BMI was then performed. The levels of cPLIN2 correlated positively with insulin and HOMA-IR, in both men and women (Table 2). Noteworthy, a strong correlation of cPLIN2 was also found with a marker of inflammation, such as leptin (Table 2). On the basis of the observed correlation with parameters related to diabetes, we checked whether people with a diagnosis of Type 2 Diabetes (T2D) have higher cPLIN2 levels than euglycemic ones. In OLD1, OLD2, OLD3 and Centenarians groups there were 36, 13, 2 and 3 subjects with T2D, respectively (Table 1). To our surprise, these subjects resulted to have cPLIN2 levels similar to those of the euglycemic subjects of the same age group (women No T2D: 47.04±27.5, T2D: 49.11±18.2; men No T2D: 26.35±21.4, T2D 31.81±17.3, cPLIN2 values are in ng/ml and expressed as mean±SD). The ANCOVA analysis considering BMI as covariate confirmed that no significant difference between the two groups is present. However, due to the small size of our study group, we cannot rule out that T2D can be a cause of increased secretion of cPLIN2.

**Table 2:** BMI-adjusted partial correlation of cPLIN2 with anthropometric, metabolic and inflammatory parameters of Cohort 1.

**Parameters** **(Correction for BMI)**	**cPLIN2** **F**		**cPLIN2** **M**	
	**Partial correlation coefficient**	**n° subjects**	**Partial correlation coefficient**	**n° subjects**
Waist circumference (cm)	-0.219	82	0.183	62
Hip circumference (cm)	0.157	80	0.234	62
Glycaemia (mg/dl)	-0.066	102	-0.205	72
Insulin (μIU/mL)	0.517***	78	0.388**	54
HOMA-IR	0.432***	78	0.295*	54
Total cholesterol (mg/dl)	0.084	102	0.075	72
HDL (mg/dl)	0.097	102	0.112	72
LDL (mg/dl)	0.192	102	-0.035	72
Triglycerides (mg/dl)	0.094	102	0.089	72
CRP (mg/dl)	-0.225	48	-0.176	37
Leptin (ng/ml)	0.606**	18	0.496*	21

Legend: ***p≤0.001; **≤0.01; *≤0.05.

Finally, in order to understand the possible interference of drug therapies on the levels of cPlin2, we compared the levels of cPLIN2 in subjects with or without lipid lowering, anti-diabetic or anti-hypertensive therapies. We analysed men and women belonging to OLD1 and OLD2 groups for which data on medications were available. As reported in Supplementary Table 1, the presence of anti-diabetic and anti-hypertensive therapies does not affect cPLIN2 levels. An apparent difference was noticed in patients undergo a lipid lowering therapy with respect to subjects that do not undergoing therapy, however after adjusting for BMI this difference resulted not statistically significant.

### cPLIN2 levels are associated with parameters of body composition

As BMI is a synthetic index of body composition, we wondered whether cPLIN2 correlates with specific parameters of fat mass (FM) or lean mass (LM). In order to deepen this aspect, we analyzed cPLIN2 levels in a group of 129 additional and well-characterized old subjects (cohort 2): 63 men and 66 women of similar age (age range: 65-79 years) from the NU-AGE Project [17]. The subjects of this cohort were selected on the basis of their BMI (range 20-30) and total FM measured by whole-body DXA scan [18, 19]. These subjects were divided into two groups: low FM and high FM (see Table 3 for FM ranges). Most of the anthropometric and biochemical measures considered were significantly different between the two FM groups, in both men and women (Table 3).

**Table 3:** Anthropometric and biochemical characteristics of Cohort 2 divided by sex and fat mass.

**Characteristics** **(mean ± SE)**	**F (N° 66)**			**M (N° 63)**		
	**Low fat mass** **(range fat mass: 14.7-22.8 kg)** **(N° 32)**	**High fat mass** **(range fat mass: 28.0-39.1 kg)** **(N° 34)**	**p** **value**	**Low fat mass** **(range fat mass: 10.6-21.5 kg)** **(N° 31)**	**High fat mass** **(range fat mass: 26.5-38.1 kg)** **(N° 32)**	**p** **value**
BMI	22.96 ± 0.27	28.04 ± 0.31	0.000	24.28 ± 0.27	28.81 ± 0.21	0.000
Waist circumference (cm)	78.39 ± 1.01	91.36 ± 0.99	0.000	89.64 ± 1.12	104.15 ± 1.09	0.000
Hip circumference (cm)	94.79 ± 0.71	106.03 ± 0.73	0.000	94.63 ± 0.77	103.72 ± 0.76	0.000
Handgrip (kg)	22.67 ± 0.82	24.04 ± 1.03	n.s.	38.65 ± 1.18	39.04 ± 1.24	n.s.
*Metabolic parameters*						
Glycaemia (mg/dl)	99.29 ± 2.19	99.15 ± 1.16	n.s.	101.06 ± 2.17	110.29 ± 3.54	0.03
Insulin (μIU/ml)	7.22 ± 0.95	9.85 ± 0.86	0.01	5.82 ± 073	13.93 ± 1.49	0.000
HOMA-IR	1.87 ± 0.33	2.43 ± 0.22	0.01	1.41 ± 0.18	3.91 ± 0.45	0.000
Total cholesterol (mg/dl)	213.69 ± 5.50	201.63 ± 5.53	n.s.	184.80 ± 6.31	181.05 ± 6.74	n.s.
HDL (mg/dl)	68.76 ± 2.93	59.64 ± 2.57	0.02	55.25 ± 2.26	47.05 ± 1.98	0.006
LDL (mg/dl)	125.89 ± 5.44	121.37 ± 4.87	n.s.	108.51 ± 5.52	110.96 ± 6.55	n.s.
Triglycerides (mg/dl)	95.19 ± 6.73	103.10 ± 6.30	n.s.	90.25 ± 6.98	119.06 ± 9.38	0.003
*Body composition parameters*						
FM (kg)	19.50 ± 0.37	31.57 ± 0.50	0.000	17.67 ± 0.57	30.59 ± 0.49	0.000
FMI (kg/m^2^)	7.89 ± 0.16	12.37 ± 0.23	0.000	6.12 ± 0.17	10.26 ± 0.15	0.000
LM (kg)	36.33 ± 0.61	39.14 ± 0.60	0.005	50.30 ± 0.88	53.31 ± 0.76	0.021
ALMI (kg/m^2^)	6.24 ± 0.10	6.73 ± 0.11	0.006	7.79 ± 0.13	8.28 ± 0.10	0.023
LMI (kg/m^2^)	14.70 ± 0.19	15.29 ± 0.19	0.045	17.48 ± 0.22	17.86 ± 0.14	n.s.
FM/LM	0.54 ± 0.01	0.81 ± 0.02	0.000	0.35 ± 0.01	0.58 ± 0.01	0.000
SMI	0.27 ± 0.003	0.24 ± 0.003	0.000	0.32 ± 0.003	0.28 ± 0.002	0.000
BMC (g)	1890.20 ± 44.75	2050.29 ± 37.22	0.006	2870.81 ± 75.79	3054.15 ± 77.48	n.s.
BMD (g/cm^2^)	0.92 ± 0.02	0.97 ± 0.01	0.01	1.17 ± 0.02	1.19 ± 0.02	n.s.
Android/gynoid FM	0.44 ± 0.03	0.56 ± 0.02	0.000	0.66 ± 0.03	0.92 ± 0.03	0.000
Android FM/LM	0.56 ± 0.03	0.98 ± 0.03	0.000	0.44 ± 0.02	0.91 ± 0.02	0.000
VAT (g)	640.21 ± 59.02	1403.18 ± 90.88	0.000	999.79 ± 76.51	2726.72 ± 119.66	0.000
SAT (g)	869.45 ± 37.97	1450.29 ± 56.46	0.000	698.07 ± 40.87	911.89 ± 74.11	0.019
**Average number of diseases**	1.57	1.5		1.6	1.62	
**Current diseases** **(n° subjects)**	diabetes (1), heart failure (8), hypercholesterolemia (16), hypertension (12), irregular heart rhythm (5), obliterative arteriopathy (8)	heart failure (4), hypercholesterolemia (18), hypertension (15), irregular heart rhythm (2), obliterative arteriopathy (12)		angina pectoris (2), diabetes (2), heart failure (8), hypercholesterolemia (14), hypertension (16), irregular heart rhythm (4), obliterative arteriopathy (4)	angina pectoris (1), diabetes (6), heart failure (7), hypercholesterolemia (11), hypertension (18), irregular heart rhythm (2), obliterative arteriopathy (7)	

Legend: FM: fat mass; FMI: fat mass index; LM: lean mass; ALMI: appendicular lean mass index; LMI: lean mass index; SMI: skeletal muscle index; BMC: bone mineral content; BMD: bone mineral density; VAT: visceral adipose tissue; SAT: subcutaneous adipose tissue; n.s. = not significant;

**Figure 1:**
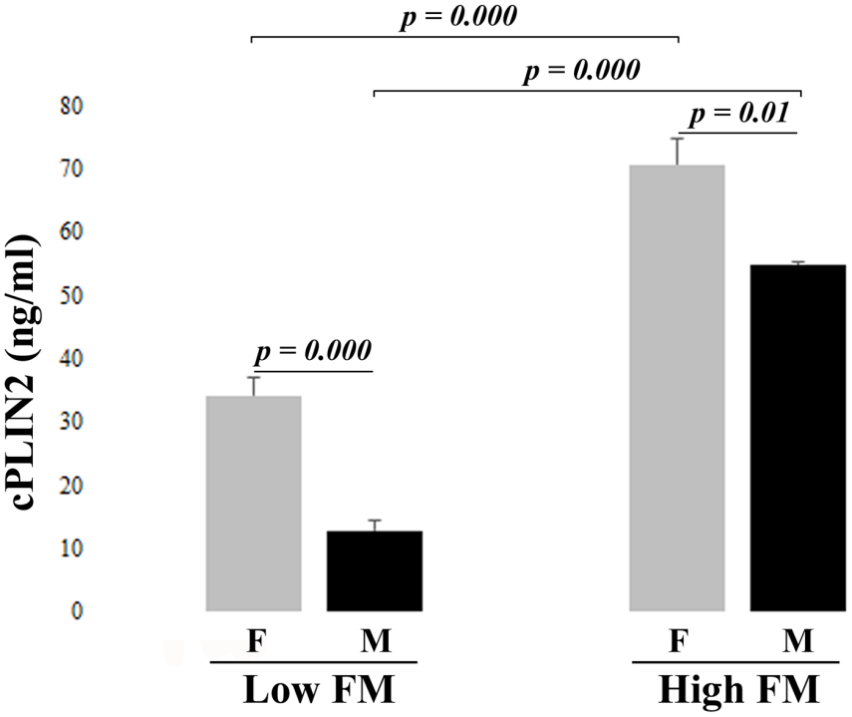
**Serum levels of cPLIN2 in subjects from cohort 2 divided by fat mass (FM): Low FM and High FM groups in women (F) and men (M), Data are expressed as mean ± SE. Significant p values < 0.05 were determined by Mann-Whitney test.**

The results of cPLIN2 in low and high FM groups are reported in Figure 1. cPLIN2 levels are significantly higher in women with respect to men, confirming the results showed in Table 1. Moreover, cPLIN2 is dramatically elevated in high FM group, in both men and women. Also in this case, since BMI strongly correlates with cPLIN2 in both women (Spearman correlation parameters: ρ =0.670, p<0.001) and men (Spearman correlation parameters: ρ =0.725, p<0.001), after adjusting for BMI, a partial correlation of cPLIN2 with anthropometric and metabolic parameters was performed (Table 4). Regarding biochemical parameters, a correlation between cPLIN2 and triglycerides is present only in women, while a weak correlation between cPLIN2 and glucose metabolism parameters (glycemia and HOMA-IR) was found only in men. This partial discrepancy with the data of Table 2 is likely due to the fact that Cohort 2 is more homogenous in terms of age range and health status [17]. Regarding body composition parameters, in both men and women positive and negative correlations were found with FM and LM parameters, respectively. Interestingly, hip circumference correlated positively in women and negatively in men, while the android/gynoid FM ratio was uncorrelated in women and positively correlated in men. A further focus on the fat of the android region indicated that the subcutaneous but not the visceral adipose tissue is strongly correlated with cPLIN2 in both sexes. Finally, a strong correlation of cPLIN2 with leptin and with alpha-1-acid glycoprotein (AGP) was found, this latter only in women (Table 4).

**Table 4:** BMI-adjusted partial correlation of cPLIN2 with anthropometric, metabolic, inflammatory and body composition parameters in Cohort 2.

**Parameters** **(Correction for BMI)**	**cPLIN2 F**		**cPLIN2 M**	
	**Partial correlation** **coefficient**	**n° subjects**	**rho**	**n° subjects**
Waist circumference	0.115	66	0.208	63
Hip circumference	0.358**	66	-0.301*	63
Glycaemia	0.175	66	0.326*	63
Insulin	0.236	66	0.201	63
HOMA-IR	0.221	66	0.288*	63
Total cholesterol	-0.077	66	-0.022	63
HDL	0.023	66	0.152	63
LDL	-0.163	66	-0.095	63
Triglycerides	0.289*	66	0.083	63
FM	0.368**	66	0.362**	63
FMI	0.488***	66	0.527***	63
LM	-0.245*	66	-0.320*	63
ALMI	-0.377**	66	-0.345**	63
LMI	-0.446***	66	-0.510***	63
FM/LM	0.466***	66	0.561***	63
SMI	-0.373**	66	-0.342**	63
BMC	-0.006	66	-0.047	63
BMD	0.086	66	0.051	63
Android/gynoid FM	-0.013	66	0.428**	63
Android FM/LM	0.348**	66	0.649***	63
VAT	-0.028	65	-0.266	56
SAT	0.471***	65	0.563***	56
Adiponectin (μg/ml)	-0.154	61	-0.102	59
AGP (mg/ml)	0.243*	61	-0.011	59
CRP (mg/dl)	0.143	61	0.046	59
Ghrelin (pg/ml)	-0.136	61	0.014	59
Leptin (ng/ml)	0.566***	61	0.566***	59
Resistin (pg/ml)	0.011	61	0.02	55

Legend: ***p≤0.001; **≤0.01; *≤0.05. FM: fat mass; FMI: fat mass index; LM: lean mass; ALMI: appendicular lean mass index; LMI: lean mass index; SMI: skeletal muscle index; BMC: bone mineral content; BMD: bone mineral density; VAT: visceral adipose tissue; SAT: subcutaneous adipose tissue; AGP: alpha-1-acid glycoprotein; CRP: C-reactive protein.

## DISCUSSION

PLIN2 is a member of the PAT family involved in the control of lipid metabolism and intracellular lipid storage and it is expressed in a variety of tissues including skeletal muscle, liver, mammary gland, heart and intestine (reviewed in [1]). Despite its exquisitely intracellular role, it has been observed that PLIN2 is also found in body fluids. In this case, its function and origin are much less clear. In the attempt to shed some light in the possible changes of circulating PLIN2 with age, in this study we have considered two cohorts of Italian subjects, one including subjects from 24 to 107 years of age, and a second one including subjects in the age range 65-79 with different levels of fat mass. We reported that the levels of cPLIN2 is higher in women with respect to men, in particular in advanced age (60-79 years) but not in old age and centenarians. This led us to test the hypothesis that such a difference was due to a different body composition. Consistently, cPLIN2 resulted strongly associated to BMI, and after correction for this parameter, all the apparent differences among age groups disappeared, thus indicating that cPLIN2 levels do not change with age. Furthermore, this led us to hypothesize that body composition parameters could be the possible drivers of cPLIN2 levels. The data obtained from Cohort 2, composed of subjects participating in the NU-AGE EU Project for which data of body composition were available, confirmed this hypothesis. In particular, cPLIN2 levels resulted positively correlated with parameters of fat mass and negatively correlated with parameters of lean mass. When considering the results related to hip circumference, android/gynoid FM and android FM/LM, it could be inferred that in women the amount but not the type of fat mass matters, while in men the android FM seems to be the parameter that drives the correlation with cPLIN2 levels. To further deepen this result, the android visceral adipose tissue (VAT) and subcutaneous adipose tissue (SAT) were considered, and only the latter results significantly correlated in both sexes. This suggests that SAT is the adipose tissue likely responsible (either directly or indirectly) of the levels of cPLIN2, and, accordingly, women have more SAT than men (as also reported in Table 3), thus likely explaining why women have higher cPLIN2 levels than men. Other possibilities to explain these sex-related differences in cPLIN2 cannot, however, be ruled out at present. As an example, mammary gland has been reported to produce PLIN2, and breast cancers characterized by elevated expression of PLIN2 result more aggressive [20, 21]. Therefore, it is also possible that at least in part the observed sex-related difference can be accounted for by the production of cPLIN2 sustained by the mammary gland.

Recently, cPLIN2 has been proposed as an adipokine [22]. However, adipose tissue does not express PLIN2 but rather PLIN1, and is not known whether it actively secretes PLIN2. On the contrary, it is well known that adipose tissue is a source of other adipokines, including pro-inflammatory leptin. Leptin is reported to be a strong inducer of the deposition of PLIN2-enriched lipid droplets in macrophages [23]. In our study, leptin is the parameter having the strongest correlation with cPLIN2 also after adjusting for BMI. Therefore, it could be hypothesized that the reason for the observed correlation between total fat mass and cPLIN2 levels may be identified in the pro-inflammatory endocrine activity played by the adipose tissue, possibly through the production of leptin. This is further, though indirectly, supported by the fact that women have higher fat mass than men [18, 24] and correspondently more circulating leptin [19, 25]. Further mechanistic experiments would be necessary to formally prove this hypothesis. Interestingly, in a previous study, we observed that inflammatory markers other than leptin such as CRP and AGP and adipose tissue-related hormones, such as adiponectin and ghrelin, are differently associated with body composition markers in old women and men [19]. It is to note, however, that none of the above-reported adipokines seemed convincingly associated with cPLIN2 levels.

We have previously reported that PLIN2 in skeletal muscle is increased in old persons, particularly in physically inactive ones [3, 5] and is associated with muscle atrophy and sarcopenia [6]. At variance with intracellular PLIN2, the levels of cPLIN2 appear to be independent of age and rather dependent on fat mass. Moreover, in skeletal muscle PLIN2 is correlated with insulin resistance [26–28]. We have observed that cPLIN2 levels are correlated with metabolic parameters such glycaemia, insulin and HOMA-IR, however, the results on this subjects were not always consistent in the two cohorts studied, and people with or without T2D resulted to have similar levels of cPLIN2, suggesting that the correlation with glycaemic parameters is probably not very strong, or masked by confounding variables. In the above-mentioned study by Fan et al. [22], a significant difference in cPLIN2 between T2D patients with or without non-alcoholic fatty liver disease (NAFLD) was reported, in particular, cPLIN2 resulted higher in T2D patients with NAFLD. However, in this study, no healthy control group is reported, and the levels of cPLIN2 are much lower than those we found in young healthy subjects, thus casting some doubts that the two measurements are comparable.

To the best of our knowledge, this is the first study describing the values of cPLIN2 in serum across the human lifespan on two cohorts of people, the first composed of subjects of different age, the latter composed of subjects with similar age but different fat mass. As a whole, despite some limitations, first of all the relatively low numerosity of the studied cohorts and their heterogeneity, some interesting data emerged from this study: i. a sex- but not age-specific difference in the levels of cPLIN2 was noticed; ii. a strong correlation with BMI and parameters of body composition emerged, in particular fat mass and SAT; iii. a strong correlation also emerged with leptin, a pro-inflammatory adipokine that could be the link between fat mass and cPLIN2 levels; iv. a more elusive and probably secondary correlation with parameters of fat and glucose metabolism also emerged that however needs further confirmation. As a final remark, we are tempting to speculate that cPLIN2 (possibly together with leptin) could be assumed as a proxy for body adiposity. Some questions remain unanswered, including the tissue origin of cPLIN2 and its biological role, which warrant further studies.

## MATERIALS AND METHODS

### Subjects

Serum samples from a total of 318 volunteers of Caucasian ancestry, in the age range from 24 to 107 years were analyzed for this study.

A first analysis was performed on 189 subjects (cohort 1), divided into five age groups: young subjects (24-39 years), OLD1 subjects (60-69 years), OLD2 subjects (70-79 years), OLD3 subjects (85-99) and Centenarians (100-107 years) enrolled in Italy in the framework of the following projects: Italian National Project PRIN09 and EU Project GEHA. The study protocols were approved by the Ethical Committee of Sant’Orsola-Malpighi University Hospital, Bologna, Italy (Ethical clearance: 118/2004/U/Tess for GEHA Project; 22/2007/U/Tess for PRIN09 Projects). All subjects signed informed consent before entering the study. Exclusion criteria were the presence of malignant neoplasia and/or current therapy with immune suppressor drugs (like cyclosporine, methotrexate, and glucocorticoids) or anticoagulant drugs. A standard questionnaire was administered to the volunteers by nurse staff in order to collect lifestyle data, health status, clinical anamnesis, and details on medications. As for centenarians, in the case that the subject was unable to respond autonomously because of hearing or sight problems, the interview was performed with a relative or a caregiver. Young subjects were free of clinically evident diseases, while, as expected, old subjects and centenarians were reported to be affected by common age-related diseases, mostly hypertension, hypercholesterolemia and diabetes (Table 1).

The second step of analyses was conducted in additional 129 well-characterized old subjects (65-79 years), 66 women and 63 men (cohort 2), divided into two groups according to their total fat mass (FM), low and high FM, measured by Dual-energy X-ray absorptiometry (DXA) (Table 3**)**. Also, in this case, the majority of the subjects were suffering from the most common age-related diseases (Table 3). These subjects are part of the Italian cohort of the EU project NU-AGE (registered with https://clinicaltrials.gov/, NCT01754012) and the recruitment of participants has been described in detail previously [17]. Briefly, volunteers aged 65-79 years from the community, free of major overt chronic diseases for at least 2 years (e.g., cancer, severe organ disease), living independently, and free of dementia, were recruited to participate in the baseline assessment. At enrollment, exclusion criteria included severe heart diseases, type 1 and insulin-treated type 2 diabetes, chronic use of corticosteroids, recent use of antibiotics, change in habitual medication use, frailty, malnutrition [body mass index (BMI) <18.5 kg/m2 or 10% weight loss within 6 months], or food allergy/intolerance requiring special diets.

Written informed consent was collected from all participants prior to their inclusion in the study, in accordance with the Declaration of Helsinki. NU-AGE was approved by the ethics committee of the S. Orsola-Malpighi Hospital Bologna, Italy (NU-AGE ethical approval n° 103/2011/U/Sper; 17/01/2012).

### Sampling and data collection

Blood was drawn in the morning after an overnight fast. All samples were processed to collect serum. Serum was obtained after clotting and centrifugation at 760g for 20 minutes at 4° C, rapidly frozen and stored at −80° C.

Total and HDL cholesterol, triglycerides, C-reactive protein (CRP), glycaemia, insulin, were measured in serum by standard biochemical assays. Insulin resistance status was assessed using the homeostasis model assessment of insulin resistance (HOMA-IR), according to the previously described formula [29]: insulin (in microunits per milliliter) × glucose (in millimoles per liter)/22.5.

Body mass index (BMI) was calculated as weight in kilograms divided by the square of the height in meters (kg/m2). Waist (WC) and hip circumference (HC) were measured using a flexible steel tape as described by Bucci and coworkers [30]. Briefly, WC was measured at the end of exhalation, by wrapping the tape at the level of the iliac crest and umbilicus, with the subject standing. HC was measured at the level of maximal protrusion of the gluteal muscles. Handgrip strength test was performed to measure the maximum isometric strength of the hand and forearm muscles using a hand-held dynamometer (SMEDLYS’ dynamometer, Scandidact, Kvistgaard, Denmark) for two performances with each hand.

Subjects included in the cohort 2 of this study underwent a whole-body DXA scan to measure total and regional body composition using the following narrow-angle fan-beam densitometer: Lunar iDXA, GE Healthcare, Madison, WI, USA – enCORETM 2011 software version 13.6; BMD CV: ≤ 1.0% (Bologna, Italy) as previously reported [18, 19]. Briefly, the region of interests included six different corporeal regions: total body, trunk, upper limbs, lower limbs, android region and gynoid region. For each region, DXA scanned the weight (in g) of total fat, lean and bone mass: whole body FM, non-bone whole body lean mass (LM), bone mineral content (BMC) and density (BMD). Measurements of visceral adipose tissue (VAT) and subcutaneous adipose tissue (SAT) were obtained at android level with CoreScan software. The relationship between parameters was investigated using specific reliable indexes: total body FM/LM, Fat Mass index (FMI, whole body fat mass/height2), Lean Mass index (LMI, whole body lean mass/height2), android/gynoid FM, android FM/LM, Appendicular Lean Mass index (ALMI, lean mass from arms plus legs/height2) and Skeletal Mass Index (SMI, lean mass from arms plus legs/weight) [31].

### Markers of inflammation, adipose related hormones and cPLIN2

Inflammatory and adipose-related markers were analyzed by a magnetic bead-based multiplex immunoassays (Bio-Plex) (Bio-Rad) according to the manufacturer’s instructions. In particular Ghrelin (inter-assay CV, 2%) and Resistin (inter-assay CV, 4%) in multiplex with Bio-Plex Pro human diabetes assay. Plates were read and analyzed by Bio-Plex Manager Software.

The quantitative determination of high sensitivity C-reactive protein (hsCRP), leptin, adiponectin has been performed by ProcartaPlexTM Immunoassay (Thermo Fisher Scientific) according to the manufacturer’s instructions. Analysis was performed using Luminex 200 instrumentation (Luminex Corporation). Assay sensitivities were 19.31 pg/mL for Leptin, 4.39 pg/mL for hsCRP, and 47.46 pg/mL for adiponectin. α1 Acid glycoprotein (AGP) has been measured by an immunoturbidimetric assay (AAGP2, Tina-quant α1-Acid Glycoprotein Gen.2 COBAS, Roche Diagnostics) with a measuring range of 0.1-4.0 g/L.

cPLIN2 concentration was determined in serum samples by ELISA assay using the commercial Human ADRP ELISA Kit (E-EL-H0278), according to the manufacturer’s instructions. After preliminary testing, serum was preferred to plasma, and samples were diluted 1:8 with the sample diluent provided by ELISA kit as this dilution resulted in the one yielding the most stable results. The intra- and inter-assay CV range were: 4.6% – 0.1% and 5.2% – 3.3%, respectively. In all the samples, PLIN2 was measured in duplicate and the mean values were used in the statistical analyses. The standard curve was determined by simultaneously analyzing a dilution series of standard samples. The final data were obtained in a blind set up by the operator. Synergy™ fluorometer (Bio-Tek Instruments, Winooski, Vermont, USA) was used to read the absorbance of each plate.

### Statistical analysis

The data were analysed with non-parametric tests since they did not follow a normal distribution. In particular, the comparison among the age groups in the cohort 1 was performed by using Kruskal-Wallis test, while Mann-Whitney test was used in cohort 1 for the comparisons between men and women, and in cohort 2 for the comparisons between low FM and high FM groups, as well as between men and women of the same FM groups. The Bonferroni correction was applied. Analysis of covariance (ANCOVA) was used to compare the mean differences in cPLIN2 levels after adjustment for BMI. The relationships between cPlin2 levels and anthropometric and biochemical parameters were calculated by Spearman rank correlation test and partial correlation analysis after adjusting for BMI. Significance was accepted as p < 0.05. Data are expressed as mean ± SE or SD. All data were analysed using the SPSS 23.0 for Windows software (SPSS Inc.; Chicago, IL, USA).

## Supplementary Material

Supplementary Table 1
