# Supplementary material for: Circulating perilipin 2 levels are associated with fat mass, inflammatory and metabolic markers and are higher in women than men
Source: Aging (Albany NY). 2021 Mar 17;13(6):7931–42. doi: 10.18632/aging.202840 (PMC8034884; doi:10.18632/aging.202840)
Supplement: Supplementary Table 1 [file aging-13-202840-s001.pdf]

Supplementary Table 1. Comparison of cPlin2 levels between subjects who take and do not take drugs for lipid lowering, anti-diabetic and anti-hypertensive therapies, in men and women, considering BMI as covariate.

	Women cPlin2 (ng/ml) – mean ± SE			Men cPlin2 (ng/ml) – mean ± SE		
	**No treat**	**Treat**	**p value**	**No treat**	**Treat**	**p value**
Lipid lowering Drugs	52.1 ± 2.7	66.0 ± 7.1	n.s.	27.5 ± 2.7	41.0 ± 8.0	n.s.
Anti-diabetic Drugs	58.1 ± 4.9	54.4 ± 3.1	n.s.	31.3 ± 4.9	30.8 ± 3.9	n.s.
Anti-hypertensive Drugs	52.4 ± 4.6	57.4 ± 3.4	n.s.	25.5 ± 3.3	35.3 ± 4.5	n.s.

n.s. = not significant.
